# Supplementary material for: Pricing Treatments Cost-Effectively when They Have Multiple Indications: Not Just a Simple Threshold Analysis
Source: Med Decis Making. 2023 Sep 12;43(7-8):914–29. doi: 10.1177/0272989X231197772 (PMC10625719; doi:10.1177/0272989X231197772)
Supplement: sj-pdf-1-mdm-10.1177_0272989X231197772 – Supplemental material for Pricing Treatments Cost-Effectively when They Have Multiple Indications: Not Just a Simple Threshold Analysis [file sj-pdf-1-mdm-10.1177_0272989X231197772.pdf]

Pricing Treatments Cost-Effectively When They Have Multiple Indications: Not Just a Simple Threshold Analysis

Supplemental Material

Jeremy D. Goldhaber-Fiebert[1] and Lauren E. Cipriano[2]

[1]Department of Health Policy and Center for Health Policy, Stanford School of Medicine and Freeman Spogli Institute, Stanford University
[2]Ivey Business School and Departments of Epidemiology & Biostatistics and Medicine, Schulich School of Medicine & Dentistry, University of Western Ontario

Corresponding author:
Lauren Cipriano, Ivey Business School Rm 2361, University of Western Ontario 1255 Western Road, London, ON, N6G 0N1, Canada.
Email: lcipriano@ivey.uwo.ca

Notation

Table S1. Table of Notation

Symbol	Description
k	Index of indications, $k \in \{0, 1, 2, ..., K\}$.
λ	Willingness to pay threshold for health benefits.
c	Marginal cost of production; $c \geq 0$.
**Parameters for each indication** k	
B_k	Per-patient expected discounted lifetime incremental health benefit for indication k.
C_k	Per-patient expected discounted lifetime incremental cost for indication k excluding the price of treatment.
q_k	Number of people eligible for indication k treatment.
$\lambda B_k - C_k$	Incremental net monetary benefit for indication k excluding the price of treatment.
**Decision variables**	
p_k	Manufacturer's decision of the price for each indication k; $p_k > c$.
d_k	Payer's decision whether or not to reimburse the treatment for indication k; $d_k \in \{0, 1\}$, where $d_k = 0$ indicates the payer does not, and where $d_k = 1$ indicates the payer does, reimburse the treatment for indication k.
**Objective functions**	
$\pi((p_k)_{\{1 \leq k \leq K\}})$	Manufacturer's profit; $\pi((p_k)_{\{1 \leq k \leq K\}}) = \sum_{k=1}^{K} d_k q_k (p_k - c)$.
$PINMB((d_k)_{\{1 \leq k \leq K\}})$	Payer's population incremental net monetary benefit; $PINMB((d_k)_{\{1 \leq k \leq K\}}) = \sum_{k=1}^{K} d_k q_k \left[\lambda B_k - (C_k + p_k)\right]$.
**Decision rules**	
$P_k^*(d_k)$	The function that maps from the payer's reimbursement decision d_k to the manufacturer's optimal pricing decision.
$D_k^*(p_k)$	A function that maps from the manufacturer's pricing decision p_k to the payer's optimal reimbursement decision.

Proofs

Social welfare maximizing policy

We formally write the optimization problem for social welfare (W)

$$\max_{(d_k)_{1\leq k\leq K}} \sum_{k=1}^{K} d_k q_k(\lambda B_k - C_k - c) \qquad \text{(OPT-W)}$$
$$\text{s.t.} \quad d_k \in \{0,1\} \quad \forall k \in \{1,\dots,K\}$$

The objective is a step function where, for each k, a non-negative component is added to the sum when $d_k = 1$ and $\lambda B_k - C_k \geq c$. Therefore, the optimal decision for each indication k, is

$$D_k^*(c) = \mathbb{1}_{\{\lambda B_k - C_k \geq c\}} \triangleq \begin{cases} 1 & \text{for } \lambda B_k - C_k \geq c \\ 0 & \text{otherwise} \end{cases}$$

□

Note on the proof strategy for the two-player games

We formally write the two connected optimization problems where the manufacturer sets the price(s) in order to maximize their profit and the payer selects the reimbursement decision(s) to maximize population incremental net monetary benefit as a bi-level optimization problem.

The solution to this problem will be a Nash equilibrium where each player selects the action that is the best response to the other player's action and there is no incentive for either player to deviate from the equilibrium solution. The optimal set of prices, $(p_k^*)_{1\leq k\leq K}$, and the optimal set of reimbursement decisions, $(d_k^*)_{1\leq k\leq K}$, represent the equilibrium solution if

$$P_k^*\left(D_k^*(p_k^*)\right) = p_k^* \qquad (1)$$

where D_k^* is the function that maps the price selected by the manufacturer to the optimal reimbursement decision by the payer, and P_k^* is the function that maps the reimbursement decision selected by the payer to the price that maximizes the manufacturer's profit.

Because the solution is an equilibrium, the bi-level optimization can be written with either the manufacturer's or the payer's problem as the outer-level optimization. We present the bi-level optimization with the manufacturer's problem in the outer-level and the payer's problem as the inner-level optimization because the exposition of the proof is slightly more straightforward in the third case. Presenting the manufacturer's problem as the outer-level optimization does not change the order of the game sequence. The formulation still takes into account the correct order of actions. The manufacturer moves first and selects price(s) that take into account the payer's best reimbursement decision as a function of the prices. The payer then makes reimbursement decisions based on observed prices. While the payer is presented as the inner-level optimization problem, the solution to that problem is a decision rule that works in response to any price decision by the manufacturer.

Case 1: Indication-Specific Prices and Reimbursement Decisions

We formally write the bi-level optimization problem for the situation where there are indication-specific prices set by the manufacturer, $(p_k)_{1\leq k\leq K}$, and indication-specific reimbursement decisions set by the payer, $(d_k)_{1\leq k\leq K}$. In this problem, the manufacturer seeks to maximize profit subject to the decisions by the payer who is seeking to maximize population incremental net monetary benefit:

$$\begin{aligned}
\max_{(p_k)_{1\leq k\leq K}} \; & \sum_{k=1}^{K} D_k^*(p_k) q_k (p_k - c) \qquad \text{(OPT1)}\\
\text{s.t.} \quad & \pi((p_k)_{1\leq k\leq K}) \geq 0\\
& p_k \geq c, \quad \forall k \in \{1,\ldots,K\}\\
& \begin{cases} \left(D_k^*(p_k)\right)_{1\leq k\leq K} = & \underset{(d_k)_{1\leq k\leq K}}{\arg\max} \sum_{k=1}^{K} d_k q_k \left[\lambda B_k - (C_k + p_k)\right]\\
& \quad \text{s.t.} \qquad PINMB((d_k)_{1\leq k\leq K}) \geq 0\\
& \qquad\qquad\quad d_k \in \{0,1\}, \quad \forall k \in \{1,\ldots,K\}
\end{cases}
\end{aligned}$$

First, we identify the feasible range of prices satisfying the payer's and the manufacturer's participation constraints. Observe that the manufacturer's participation constraint, $\pi((p_k)_{1\leq k\leq K}) \geq 0$, where

$$\pi((p_k)_{1\leq k\leq K}) = \sum_{k=1}^{K} d_k q_k (p_k - c),$$

is linear and increasing in p_k, for each k, when $d_k = 1$, adding a non-negative component to the sum whenever $p_k \geq c$. Also observe that the payer's participation constraint, $PINMB((d_k)_{1\leq k\leq K}) \geq 0$, where

$$PINMB((d_k)_{1\leq k\leq K}) = \sum_{k=1}^{K} d_k q_k \left[\lambda B_k - (C_k + p_k)\right],$$

is linear and decreasing in p_k, for each k, when $d_k = 1$, adding a non-negative component to the sum whenever $p_k \leq \lambda B_k - C_k$. Combining these participation constraints, for each indication, feasible prices exist where $\lambda B_k - C_k \geq p_k \geq c$.

Second, we identify the optimal solution for each of the optimization problems. The payer's optimization problem is the inner problem

$$\begin{aligned}
\underset{(d_k)_{1\leq k\leq K}}{\arg\max} \; & \sum_{k=1}^{K} d_k q_k \left[\lambda B_k - (C_k + p_k)\right]\\
\text{s.t.} \quad & PINMB((d_k)_{1\leq k\leq K}) \geq 0\\
& d_k \in \{0,1\} \quad \forall k \in \{1,\ldots,K\}
\end{aligned}$$

Population incremental net monetary benefit is maximized by providing reimbursement for any indication where $\lambda B_k - C_k$ is greater than or equal to the offered price. Therefore,

$$D_k^*(p_k) = \mathbb{1}_{\{\lambda B_k - C_k \geq p_k\}} \triangleq \begin{cases} 1 & \text{for } \lambda B_k - C_k \geq p_k \\ 0 & \text{otherwise} \end{cases}$$

Substituting $D_k^*(p_k)$ into the outer maximization problem (i.e., the manufacturer's problem) will identify the manufacturer's best response to the payer's optimal action:

$$\max_{(p_k)_{1 \leq k \leq K}} \sum_{k=1}^{K} \mathbb{1}_{\{\lambda B_k - C_k \geq p_k\}} q_k (p_k - c)$$
$$p_k \geq c \quad \forall k \in \{1, \ldots, K\}$$

The manufacturer's profit is maximized at the largest feasible p_k, for each k, because the manufacturer's profit is linear and increasing in each p_k. Therefore,

$$p_k^* = \begin{cases} \lambda B_k - C_k & \text{for } \lambda B_k - C_k \geq c \\ c & \text{otherwise} \end{cases}$$

which is the fixed point defined in Eq. (1). In words, this identifies the best price decision for the manufacturer, meeting the objective of maximizing profit, taking into account the payer's best reimbursement decision in response to manufacturer's price. □

In the general case, this creates 2^K possible policy regions, including all possible combinations of reimbursement or not for each indication. Ultimately, for each indication where $\lambda B_k - C_k \geq c$, the manufacturer prices at the maximum acceptable price for the payer, and the manufacturer captures all of the surplus. For the case of $K = 2$, the policy outcome of this optimization problem is illustrated in Figure 1A.

Case 2: Single price and single reimbursement decision for multiple indications

We formally write the bi-level optimization problem for the situation where there is a single price set by the manufacturer, $p \geq c$, and a single reimbursement decision set by the payer, $d \in \{0, 1\}$. In this problem, the manufacturer seeks to maximize profit subject to the decisions by the payer who is seeking to maximize population incremental net monetary benefit:

$$\begin{aligned} \max_p \sum_{k=1}^{K} & D_k^*(p) q_k (p - c) \qquad \text{(OPT2)} \\ \text{s.t.} \quad & \pi(p) \geq 0 \\ & p \geq c \\ & \begin{cases} D_k^*(p) = & \arg\max_d \sum_{k=1}^{K} d q_k \left[\lambda B_k - (C_k + p)\right] \\ & \quad \text{s.t.} \qquad PINMB(d) \geq 0 \\ & \qquad\qquad\quad d \in \{0, 1\} \end{cases} \end{aligned}$$

First, we identify the feasible range of prices satisfying the payer's and the manufacturer's participation constraints. Observe that the manufacturer's participation constraint, $\pi(p) \geq 0$, where

$$\pi(p) = \sum_{k=1}^{K} d q_k (p - c),$$

is linear and increasing in p when $d = 1$, adding a non-negative component to the sum whenever $p \geq c$. Also observe that the payer's participation constraint, $PINMB(d) \geq 0$, where

$$PINMB(d) = \sum_{k=1}^{K} d q_k \left[\lambda B_k - (C_k + p)\right],$$

is linear and decreasing in p when $d = 1$. Setting $d = 1$ and re-arranging the payer's participation constraint reveals the payer's maximum acceptable price, which we denote $\bar{p}$, to be

$$\bar{p} = \frac{\sum_{k=1}^{K} q_k \left[\lambda B_k - C_k\right]}{\sum_{k=1}^{K} q_k}.$$

Combining these participation constraints, for each indication, feasible prices exist where $\bar{p} \geq p \geq c$.

Second, we identify the optimal solution for each of the optimization problems. The payer's optimization problem is the inner problem

$$\begin{aligned} \arg\max_d \sum_{k=1}^{K} & d q_k \left[\lambda B_k - (C_k + p)\right] \\ \text{s.t.} \quad & PINMB(d) \geq 0 \\ & d \in \{0, 1\} \end{aligned}$$

Population incremental net monetary benefit is maximized by providing reimbursement, i.e., $d = 1$, when the weighted average incremental net monetary benefit excluding the price of treatment is greater than or equal to the offered price. Therefore,

$$D^*(p) = \mathbb{1}_{\{p \leq \bar{p}\}} \triangleq \begin{cases} 1 & \text{for } p \leq \bar{p} = \frac{\sum_{k=1}^{K} q_k[\lambda B_k - C_k]}{\sum_{k=1}^{K} q_k} \\ 0 & \text{otherwise} \end{cases}$$

Substituting $D^*(p)$ into the outer maximization problem will identify the manufacturer's best response to the payer's optimal action:

$$\max_p \sum_{k=1}^{K} \mathbb{1}_{\{p \leq \bar{p}\}} q_k(p - c)$$
$$p \geq c$$

The manufacturer's profit is maximized at the largest feasible p because the manufacturer's profit is linear and increasing in each p. Therefore,

$$p^* = \begin{cases} \bar{p} & \text{for } \frac{\sum_{k=1}^{K} q_k[\lambda B_k - C_k]}{\sum_{k=1}^{K} q_k} \geq c \\ c & \text{otherwise} \end{cases}$$

which is the fixed point defined in Eq. (1). Again, this identifies the best price decision for the manufacturer, meeting the objective of maximizing profit, taking into account the payer's best reimbursement decision in response to manufacturer's price. □

This creates 2 possible policy regions: one in which the payer provides reimbursement for all indications at the manufacturer's offered price of $\bar{p}$ and one in which the payer provides no reimbursement for any indication. Overall, all the surplus is taken by the manufacturer (consumer surplus is zero). For the case of $K = 2$, the policy outcome of this optimization problem is illustrated in Figure 1B.

Case 3: Single price with indication-specific reimbursement decisions

We formally write the bi-level optimization problem for the situation where there is a single price set by the manufacturer, $p \geq c$, and indication specific reimbursement decisions set by the payer, $(d_k)_{1\leq k\leq K}$. To find the solution to this problem, it is useful to rank the potential indications based on their incremental net monetary benefit excluding the price of treatment, i.e., $\lambda B_k - C_k$. So, without loss of generality, we assume that indications are ranked such that $k = 1$ for the highest valued indication, $k = 2$ for the second-highest valued indication, and $k = K$ for the lowest value indication. The sequenced list can then be written

$$\lambda B_K - C_K \leq \lambda B_{K-1} - C_{K-1} \leq ... \leq \lambda B_2 - C_2 \leq \lambda B_1 - C_1.$$

Let $n \leq K$ represent the number of indications where $\lambda B_k - C_k \geq c$.

The manufacture seeks to maximize profit subject to the decisions by the payer who is seeking to maximize population net monetary benefit:

$$\begin{aligned} \max_p & \sum_{k=1}^{K} D_k^*(p) q_k (p - c) \qquad \text{(OPT3)} \\ \text{s.t.} \quad & \pi(p) \geq 0 \\ & p \geq c \\ & \begin{cases} \left(D_k^*(p)\right)_{1\leq k\leq K} = & \underset{(d_k)_{1\leq k\leq K}}{\arg\max} \ \sum_{k=1}^{K} d_k q_k \left[\lambda B_k - (C_k + p)\right] \\ & \text{s.t.} \qquad PINMB((d_k)_{1\leq k\leq K}) \geq 0 \\ & \qquad\qquad d_k \in \{0,1\}, \quad \forall k \in \{1,\dots,K\} \end{cases} \end{aligned}$$

First, we show that the manufacturer can limit their decision alternatives to $n + 1$ possible pricing decisions each representing the selection of a threshold price where $p = \lambda B_k - C_k$ for some $k \leq n$, plus the possible decision to choose the reserve price, c, the marginal cost of production. Consider an offered price $p = p'$ where $p' = \lambda B_i - C_i > c$. The payer's optimal action will be $d_k = 1$ for all indications where $k \leq i$ because each of these indications will contribute a non-negative component to the sum in the payer's objective function. The payer will not reimburse for any indication where $k > i$, because $\lambda B_k - C_k - p' < 0$ for $k > i$, which would contribute a negative component to the sum in the payer's objective function. Next, consider an offered price $p = p''$ where $\lambda B_i - C_i > p'' > \lambda B_{i+1} - C_{i+1} > c$. Again, the payer's optimal action will be $d_k = 1$ for all indications where $k \leq i$ because each of these indications will contribute a non-negative component to the sum in the payer's objective function. The payer will choose $d_{i+1} = 0$ because $\lambda B_{i+1} - C_{i+1} - p'' < 0$, and, similarly, will choose $d_k = 0$ for all $k > i$. So, to achieve the policy outcome of $d_k = 1$ for $k \leq i$ and $d_k = 0$ for $k > i$, the manufacturer could offer either price $p' = \lambda B_i - C_i$ or a lower price, p'', where $\lambda B_i - C_i > p'' > \lambda B_{i+1} - C_{i+1} > c$. Conditional on the policy outcome, the manufacturer's objective function is strictly increasing in p, therefore, the manufacturer would always choose to offer p', not p''. Therefore, the manufacturer only

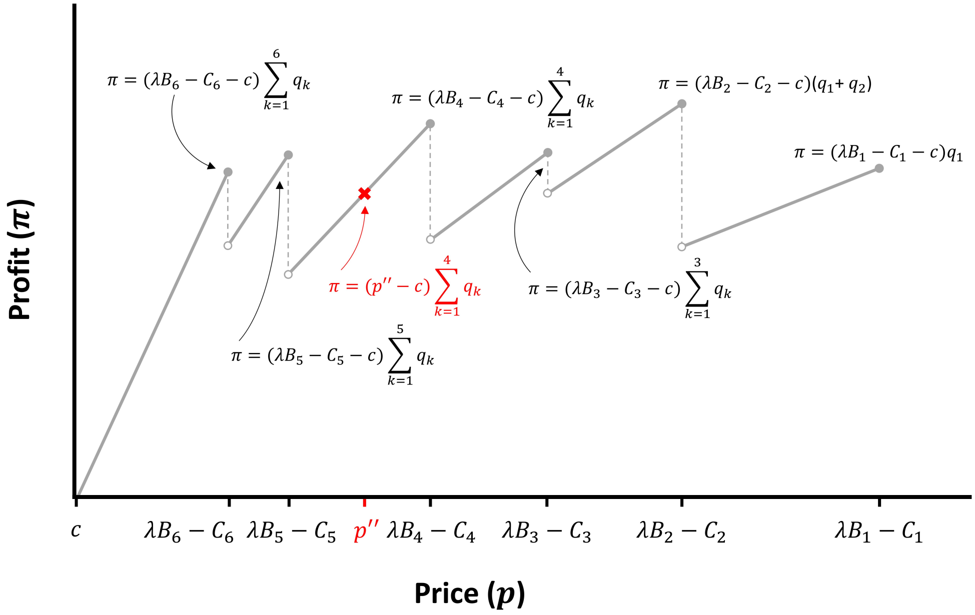

Figure S1. Manufacturer profit (π) at various manufacturer selected prices and quantities resulting from corresponding payer reimbursement decisions. The red $\times$ represents the manufacturer's profit if they choose price p'', where $\lambda B_5 - C_5 \leq p'' \leq \lambda B_4 - C_4$, resulting in the payer reimbursing for all indications $k \leq 4$. Compared to this price, the manufacturer can make more profit by offering the higher price $p = \lambda B_4 - C_4$ which leads to the same policy decision (and, therefore, quantity of sales).

needs to consider the set of prices exactly equal to the net incremental benefit excluding treatment cost for each indication. We illustrate the dominance of the set of prices equal to the population incremental net monetary benefit of each indication over any intermediate price in Supplemental Figure S1.

Similarly, the payer can also limit their decision space to $n + 1$ possible policy decisions each representing the selection of a threshold k below which all indications are reimbursed and none above it, plus the potential decision to not reimburse any indications.

Second, we identify the optimal solution for each of the optimization problems. The payer's optimization problem is the inner problem.

$$\begin{aligned}
\underset{(d_k)_{1\leq k\leq K}}{\arg\max} \sum_{k=1}^{K} d_k q_k \left[\lambda B_k - (C_k + p)\right] \\
\text{s.t.} \quad PINMB((d_k)_{1\leq k\leq K}) \geq 0 \\
d_k \in \{0,1\} \quad \forall k \in \{1,\ldots,K\}
\end{aligned}$$

Population incremental net monetary benefit is maximized by providing reimbursement for any indication where $\lambda B_k - C_k$ is greater than or equal to the offered price. Therefore,

$$D_k^*(p) = \mathbb{1}_{\{\lambda B_k - C_k \geq p\}} \triangleq \begin{cases} 1 & \text{for } \lambda B_k - C_k \geq p \\ 0 & \text{otherwise} \end{cases}$$

Substituting $D_k^*(p)$ into the manufacturer's optimization problem will identify the manufacturer's best response to the payer's optimal action:

$$\begin{aligned} &\max_p \sum_{k=1}^{K} \mathbb{1}_{\{\lambda B_k - C_k \geq p\}} q_k(p-c) \\ &\text{s.t. } p \in \{(\lambda B_1 - C_1), (\lambda B_2 - C_2), ..., (\lambda B_n - C_n), c\} \end{aligned}$$

To identify the optimal price(s), the objective function needs to be computed for each possible price (at most $K+1$ possible prices). The price(s) that maximize profit will satisfy the fixed point in Eq. 1. □

We note that there is no guarantee of a single optimal solution in the general case (there may be two or more prices that, by chance, lead to the exact same optimal profit). However, even if there are multiple equilibrium points, each equilibrium point will be stable with no incentive for either party to deviate.

This solution creates up to $K+1$ possible policy regions where the manufacturer may select a price such that the payer chooses to reimburse a subset of the possible indications. To gain insight into the features of the policy space, we illustrate that the manufacturer's optimal price requires the manufacturer to balance the opportunity for a larger sales volume when they select a lower price. Consider two prices $p_m > p_{m+1} > c$ where $p_m = \lambda B_m - C_m$ and $p_{m+1} = \lambda B_{m+1} - C_{m+1}$. If the manufacturer offers price p_m, the payer will reimburse for all indications $k \leq m$ and the manufacturer's profit will be

$$\begin{aligned} \pi(p_m = \lambda B_m - C_m) &= \sum_{k=1}^{K} D_k^*(p_m) q_k(p_m - c) \\ &= \sum_{k=1}^{m} q_k(p_m - c) \end{aligned}$$

This is represented by the red rectangle in Figure S2 (areas A and C). If the manufacturer offers price p_{m+1}, the payer will reimburse for all indications $k \leq m+1$ and the manufacturer's profit will be

$$\begin{aligned} \pi(p_{m+1} = \lambda B_{m+1} - C_{m+1}) &= \sum_{k=1}^{K} D_k^*(p_{m+1}) q_k(p_{m+1} - c) \\ &= \sum_{k=1}^{m+1} q_k(p_{m+1} - c) \end{aligned}$$

This is represented by the blue rectangle in Figure S2 (areas B and C).

The manufacturer prefers the lower price, p_{m+1}, when doing so generates additional profit through a larger volume of sales compensating for the lost revenue associated with the lower price on all sales:

$$\pi(p_{m+1}) \geq \pi(p_m)$$
$$\sum_{k=1}^{m+1} q_k(p_{m+1} - c) \geq \sum_{k=1}^{m} q_k(p_m - c)$$
$$(p_{m+1} - c)q_{m+1} \geq (p_m - p_{m+1}) \sum_{k=1}^{m} q_k \quad (2)$$

The left side of this inequality represents additional profit from an increase in sales quantity and is illustrated as the blue rectangle (area B only). The right side of this inequality represents a loss in profit associated with offering a lower price (in order to obtain a larger quantity of sales) and is illustrated as the red rectangle (area A only) in Supplemental Figure S2.

Re-writing Eq. 2 provides insights into the conditions leading to this preference for a lower price (and higher volume):

$$\frac{q_{m+1}}{\sum_{k=1}^{m} q_k} \geq \frac{(p_m - p_{m+1})}{(p_{m+1} - c)}$$

The left hand side represents the percent change in quantity sold by selecting the lower price and the right hand side represents the percent change in profit margin when selecting the higher price. If the percent increase in quantity sold when selecting the lower price exceeds the percent change in margin when selecting the higher price, the manufacturer prefers the lower price in their effort to maximize profit.

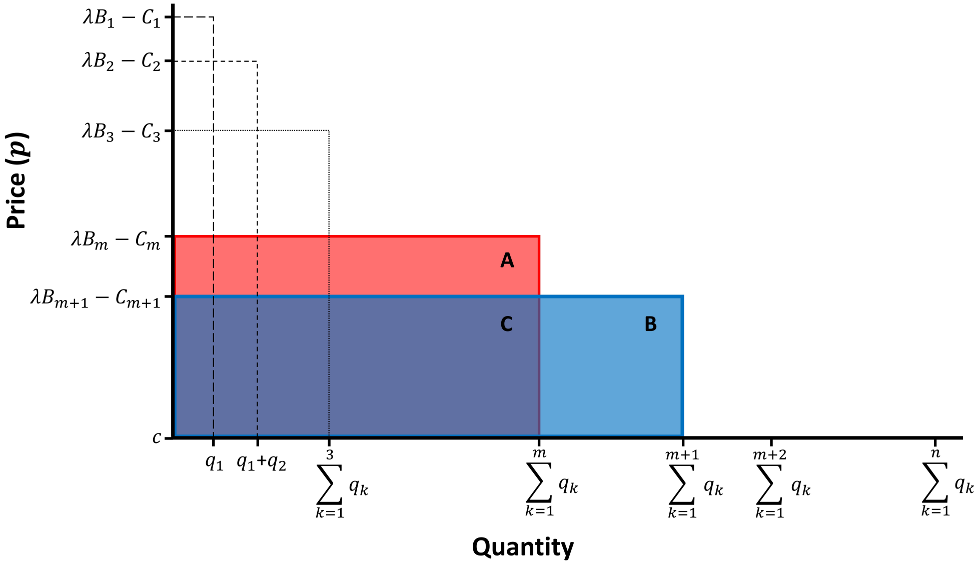

Figure S2. Manufacturer profit (π) at various manufacturer selected prices and quantities resulting from corresponding payer reimbursement decisions. The red rectangle represents the manufacturer's profit if they choose price $p_m = \lambda B_m - C_m$, which will result in the payer reimbursing for all indications $k \leq m$. The blue rectangle represents the manufacturer's profit if they choose price $p_{m+1} = \lambda B_{m+1} - C_{m+1}$, which will result in the payer reimbursing for all indications $k \leq m$.

Case study

As an illustrative exercise, we used our method to assess the optimal price for drugs with multiple indications as described in Bach (2014).[1] With permission, we have reproduced the main information from the analysis in Bach (2014) in Supplemental Table S2.

Bach (2014) considered 4 drugs that have 2-3 indications each. For each indication, Bach (2014) presents the treatment efficacy in terms of median survival gain, typical treatment duration, typical total treatment cost, current monthly price, and the treatment cost per increase in median life expectancy. The table also presents the hypothetical monthly treatment cost under two potential frameworks for pricing: (1) if pricing was anchored to the indication with the most value, and (2) if pricing was indication-specific and valued on a $150,000 per year-of-life-gained threshold.

To apply the outcomes of the game sequences to these examples, we first need to estimate the eligible population sizes for each indication. We then need to calculate incremental net monetary benefit excluding the drug price.

Estimating the eligible population size

We present rough estimates of patient populations for each indication relevant to each drug in the United States in Supplemental Table S3. The 'Major cancer type' column shows total annual US incidence for the latest years available for major cancer type like 'lung cancer' or 'breast cancer'. The proportion of patients in the specific subcategory column reports the fraction of the individuals in each major cancer type that pertain to the specific population targeted by one of the drug's indications based on patient distribution/classification at diagnosis. For example, approximately 6% of incident breast cancers are metastatic. Likewise, approximately 85% of lung cancers are non-small-cell. The key feature to notice about this data is that the size of the patient populations for a specific drug such as nab-Paclitaxel (Abraxane) can be very different, ranging from less than 16,000 breast cancer patients to over 200,000 non-small-cell lung cancer patients.

Estimating the net monetary benefit excluding drug price

To compute the incremental net monetary benefit excluding drug price, we must convert other information provided by Bach (2014) into appropriate units for our analysis.

We crudely convert their reported median gains in survival expressed in life years into gains in quality-adjusted life-years (QALYs). First, median survival gains are converted to mean survival gains under the assumption of exponential hazards by dividing them by $(-ln(0.5))$. Second, we assume that for these generally advanced or metastatic cancers the health-related quality of life weight is 0.4. We therefore multiply the average life expectancy gains by 0.4 to compute quality-adjusted life expectancy gains. As survival is relatively short, and this exercise is done for illustrative purposes, we do not apply discounting.

Finally, we convert these health gains into monetary equivalents using a willingness-to-pay (WTP) threshold of $150,000 per QALY-gained. As a simplification, we also assume that the additional survival gained does not produce substantial additional medical costs beyond the cost of the drug. Hence, we can compute per-patient net monetary benefit exclusive of drug price for each indication for each drug (Supplemental Table S4). The key feature to notice about the incremental net monetary benefit excluding drug price is that there is large variation across indications for a specific drug. For example, for Erlotinib (Tarceva) the incremental net monetary benefit excluding the cost of treatment is $24,237 for patients with non-small cell lung cancer and $2,597 for patients with pancreatic cancer.

Table S2. Original Data from Bach (2014).[1] Monetary values are presented in 2014 US$.

Drug and indication	Median survival gain (y)	Typical treatment duration (months)	Typical treatment cost	Cost per Year of Life gained (median)	Current monthly price	Monthly price based on indication with most value	Monthly price based on achieving $150K per LYG
**nab-Paclitaxel (Abraxane)**							
Metastatic breast cancer	0.185	4.167	25,990	145,288	6,255	6,255	6,458
Non–small-cell lung cancer	0.080	4.160	29,988	399,840	7,217	2,622	2,708
Pancreatic cancer	0.150	4.000	27,065	180,433	6,766	5,448	5,625
**Erlotinib (Tarceva)**							
First line treatment of metastatic non small-cell lung cancer	0.280	8.200	51,596	182,104	6,292	6,292	5,183
Pancreatic cancer	0.030	3.906	21,696	650,885	5,563	1,556	1,282
**Cetuximab (Erbitux)**							
Locally advanced squamous cell carcinoma of the head and neck	1.640	1.397	14,292	8,706	10,319	10,319	177,798
First-line treatment of recurrent or metastatic squamous cell carcinoma of the head and neck	0.230	4.160	42,875	190,556	10,319	471	8,123
**Trastuzumab (Herceptin)**							
Adjuvant treatment of breast cancer	1.998	12.000	64,941	32,645	5,412	5,412	24,867
Metastatic breast cancer	0.400	10.000	54,118	135,294	5,412	905	6,000

Table S3. Rough Estimates of Patient Population Sizes

	Major cancer type	Proportion of patients in specific subcategory	Total annual incident cases
**nab-Paclitaxel (Abraxane)**			
Metastatic breast cancer	264,000	6%	15,840
Non–small-cell lung cancer	238,340	85%	202,589
Pancreatic cancer	64,050	100%	54,443
**Erlotinib (Tarceva)**			
First line treatment of metastatic non small-cell lung cancer	202,589	30%	60,777
Pancreatic cancer	64,050	100%	54,443
**Cetuximab (Erbitux)**			
Locally advanced squamous cell carcinoma of the head and neck	66,920	50%	33,460
First-line treatment of recurrent or metastatic squamous cell carcinoma of the head and neck	66,920	10%	6,692
**Trastuzumab (Herceptin)**			
Adjuvant treatment of breast cancer	264,000	24%	63,360
Metastatic breast cancer	264,000	6%	15,840

Analysis

Indication-specific pricing and reimbursement decisions

Under the “indication-specific pricing and reimbursement decisions” game, the total indication-specific price for each drug will be equal to the per-patient net monetary benefit exclusive of drug prices. In the main paper, we made the simplification that the price of the treatment was paid all at once and upfront. However, Bach (2014) reports typical treatment times expressed in months. If “typical” implies average, then we can compute the indication-specific per-month price for each drug by dividing the total indication-specific price by the duration of treatment (Supplemental Table S4).

Single price and single reimbursement decision

Under the “single price and reimbursement decision” game, the size of the patient population becomes relevant. But as different patient populations are treated for different lengths of time and treatment is provided repeatedly (e.g., monthly treatment purchase), the weighting function to determine the single price uses the relative number of patient-months for each indication instead of the relative number of patients for each indication (Supplemental Table S4).

Single price and indication-specific reimbursement decisions

Under the “single price with indication-specific reimbursement decisions” game, the manufacturer chooses the monthly price that maximizes total profit. Without knowledge of the marginal cost of production, we use revenue as a proxy for profit for illustrative purposes. We calculate profit as the sum over all reimbursed indications [monthly price × indication-specific incidence × indication-specific treatment duration]. The manufacturer will select a price equal to the threshold price for one of the indications and the payer will only reimburse indications providing as much or more value than that indication. The results are also shown in Supplemental Table S4.

Comparison across games

As shown in Supplemental Table S4, in the first game, each indication for each drug is reimbursed at a distinct price corresponding to the value it produces. In the second game, the monthly price is a linear combination of the prices from the first game with subsidization of value across indications. In both of these games all indications are reimbursed for each drug and the price is set such that all of the surplus is captured by the manufacturer in the form of profits; there is no consumer surplus for the payer.

In the third game, for three of the four example drugs, the payer would decide not to reimburse all indications at the profit-maximizing price offered by the manufacturer. The indications that are not reimbursed generate less value in terms of health benefits for patients and hence require the manufacturer to offer a lower price. In these examples, the outcome is not the first-best outcome (in which all patients receive treatment). The manufacturer earns less profit than it did in games 1 and 2. There is also no consumer surplus because the manufacturer has chosen to price at the highest price obtaining sales for only the highest-value indication.

For one of the drugs in the example, nab-Paclitaxel (Abraxane), the manufacturer’s profit maximizing price is the threshold price for the lowest-value indication. This price offer leads the payer to reimburse for all indications. In this scenario, the first-best outcome is obtained (all patients receive treatment). Even though social welfare is the same, the manufacturer earns less profit than in games 1 and 2, because there is positive consumer surplus.

Table S4. Monthly Pricing and Reimbursement under Each Game

		Game 1		Game 2		Game 3	
	INMB w/o drug costs	Monthly price	Total price	Monthly price	Total price	Reimbursed (Y/N)	Monthly Price
**nab-Paclitaxel (Abraxane)**							
Metastatic breast cancer	$16,014	$3,843	$16,014	$2,098	$8,742	Y	$1,665
Non–small-cell lung cancer	$6,925	$1,665	$6,925	$2,098	$8,728	Y	$1,665
Pancreatic cancer	$12,984	$3,246	$12,984	$2,098	$8,392	Y	$1,665
**Erlotinib (Tarceva)**							
First line treatment of metastatic non small-cell lung cancer	$24,237	$2,956	$24,237	$2,271	$18,619	Y	$2,956
Pancreatic cancer	$2,597	$665	$2,597	$2,271	$8,869	N	na
**Cetuximab (Erbitux)**							
Locally advanced squamous cell carcinoma of the head and neck	$141,961	$101,619	$141,961	$65,475	$91,468	Y	$101,619
First-line treatment of recurrent or metastatic squamous cell carcinoma of the head and neck	$19,909	$4,786	$19,909	$65,475	$272,375	N	na
**Trastuzumab (Herceptin)**							
Adjuvant treatment of breast cancer	$172,950	$14,413	$172,950	$12,525	$150,295	Y	$14,413
Metastatic breast cancer	$34,625	$3,462	$34,625	$12,525	$125,246	N	na

References

[1] Bach PB. Indication-specific pricing for cancer drugs. JAMA. 2014;312(16):1629-30.
